# Supplementary material for: Short-term caloric restriction or resveratrol supplementation alters large-scale brain network connectivity in male and female rats
Source: Front Nutr. 2025 Feb 3;12:1440373. doi: 10.3389/fnut.2025.1440373 (PMC11830597; doi:10.3389/fnut.2025.1440373)
Supplement: Supplementary file 1 [file Table_1.DOCX]

Supplementary Material

**Supplementary Table 1.** Brain regions and Network abbreviations

| Network | Region | Abbreviation |
| --- | --- | --- |
| Default mode-like network (DMLN) | Cingulate cortex left  Cingulate cortex right  Retrosplenial cortex left  Retrosplenial cortex right  Prelimbic cortex left  Prelimbic cortex right  Temporal Association left  Temporal Association right | Cg L  Cg R  RSC L  RSC R  Limbic L  Limbic R  TeA L  TeA R |
| Hippocampal network (Hipp) | Hippocampal field CA1 left  Hippocampal field CA1 right  Hippocampal field CA3 left  Hippocampal field CA3 right  Dentate gyrus left  Dentate gyrus right  Entorhinal cortex left  Entorhinal cortex right | CA1 L  CA1 R  CA3 L  CA3 R  DG L  DG R  Ent L  Ent R |
| Sensory network (Sens) | Visual cortex left  Visual cortex right  Auditory cortex left  Auditory cortex right  Piriform cortex left  Piriform cortex right | VC L  VC R  Aud L  Aud R  Pir L  Pir R |
| Lateral cortical network (LCN) | Primary somatosensory cortex left  Primary somatosensory cortex right  Secondary somatosensory cortex left  Secondary somatosensory cortex right  Motor cortex left  Motor cortex right  Frontal association cortex left  Frontal association cortex right  Insular cortex left  Insular cortex right | S1 L  S1 R  S2 L  S2 R  MC L  MC R  FrA L  FrA R  Ins L  Ins R |
| Subcortical network (SubC) | Caudate putamen left  Caudate putamen right  Nucleus Accumbens left  Nucleus Accumbens right  Basal forebrain  Thalamus left  Thalamus right  Medial septum left  Medial septum right  Hypothalamus left  Hypothalamus right | CPu L  CPu R  NAcc L  NAcc R  BFB  Thal L  Thal R  MS L  MS R  Hyp L  Hyp R |

**Supplementary Table 2.** Statistical outcomes of the one-sample t-test analysis for the males of each within and between network zFC per treatment.

|  | **One-sample t-test** | | | | | | | |
| --- | --- | --- | --- | --- | --- | --- | --- | --- |
| **Network** | **Males** | | | | | | | |
|  | **Ctrl** | | **Rsv** | | | **CR** | | |
|  | *FC mean* ± *SD* | *FDR corrected*  *p-value* | *FC mean* ± *SD* | *FDR corrected*  *p-value* | *FC mean* ± *SD* | | *FDR corrected*  *p-value* |  |
| **DMLN-DMLN** | 0.497 ± 0.117 | 0.0029* | 0.379 ± 0.094 | <0.0001* | 0.527 ± 0.268 | | 0.0126* |  |
| **DMLN-Hipp** | 0.401 ± 0.108 | 0.0029* | 0.290 ± 0.076 | <0.0001* | 0.391 ± 0.170 | | 0.0079* |  |
| **DMLN-Sens** | 0.491 ± 0.130 | 0.0029* | 0.352 ± 0.119 | <0.0001* | 0.505 ± 0.211 | | 0.0076* |  |
| **DMLN-LCN** | 0.269 ± 0.081 | 0.0029* | 0.187 ± 0.076 | <0.0001* | 0.301 ± 0.163 | | 0.0147* |  |
| **DMLN-SubC** | 0.1 ± 0.062 | 0.004* | 0.134 ± 0.033 | <0.0001* | 0.199 ± 0.066 | | 0.0076* |  |
| **Hipp-Hipp** | 0.355 ± 0.111 | 0.0029* | 0.279 ± 0.071 | <0.0001* | 0.328 ± 0.138 | | 0.0076* |  |
| **Hipp-Sens** | 0.414 ± 0.127 | 0.0029* | 0.314 ± 0.086 | <0.0001* | 0.406 ± 0.142 | | 0.0070* |  |
| **Hipp-LCN** | 0.265 ± 0.084 | 0.0029* | 0.207 ± 0.062 | <0.0001* | 0.295 ± 0.113 | | 0.0076* |  |
| **Hipp-SubC** | 0.150 ± 0.059 | 0.0055* | 0.124 ± 0.022 | <0.0001* | 0.169 ± 0.053 | | 0.0070* |  |
| **Sens-Sens** | 0.564 ± 0.190 | 0.0033* | 0.415 ± 0.120 | <0.0001* | 0.570 ± 0.178 | | 0.0070* |  |
| **Sens-LCN** | 0.362 ± 0.106 | 0.0029* | 0.283 ± 0.072 | <0.0001* | 0.394 ± 0.160 | | 0.0076* |  |
| **Sens-SubC** | 0.180 ± 0.061 | 0.0043* | 0.149 ± 0.031 | <0.0001* | 0.194 ± 0.062 | | 0.0070* |  |
| **LCN-LCN** | 0.469 ± 0.065 | 0.0001* | 0.413 ± 0.043 | <0.0001* | 0.521 ± 0.212 | | 0.0076* |  |
| **LCN-SubC** | 0.147 ± 0.043 | 0.0028* | 0.125 ± 0.035 | <0.0001* | 0.175 ± 0.061 | | 0.0070* |  |
| **SubC-SubC** | 0.113 ± 0.037 | 0.003* | 0.093 ± 0.024 | <0.0001* | 0.136 ± 0.037 | | 0.0076* |  |

**Supplementary Table 3.** Statistical outcomes of the one-sample t-test analysis for the females of each within and between network zFC per treatment.

|  | **One-sample t-test** | | | | | |
| --- | --- | --- | --- | --- | --- | --- |
| **Network** | **Females** | | | | | |
|  | **Ctrl** | | **Rsv** | | **CR** | |
|  | *FC mean* ± *SD* | *FDR corrected*  *p-value* | *FC mean* ± *SD* | *FDR corrected*  *p-value* | *FC mean* ± *SD* | *FDR corrected*  *p-value* |
| **DMLN-DMLN** | 0.620 ± 0.149 | 0.0002* | 0.482 ± 0.174 | <0.0001* | 0.473 ± 0.134 | <0.0001* |
| **DMLN-Hipp** | 0.455 ± 0.117 | 0.0002* | 0.370 ± 0.108 | <0.0001* | 0.380 ± 0.056 | <0.0001* |
| **DMLN-Sens** | 0.565 ± 0.121 | <0.0001* | 0.464 ± 0.099 | <0.0001* | 0.456 ± 0.120 | <0.0001* |
| **DMLN-LCN** | 0.359 ± 0.040 | <0.0001* | 0.286 ± 0.079 | <0.0001* | 0.245 ± 0.093 | 0.0006* |
| **DMLN-SubC** | 0.240 ± 0.048 | <0.0001* | 0.171 ± 0.042 | <0.0001* | 0.179 ± 0.061 | 0.0002* |
| **Hipp-Hipp** | 0.413 ± 0.096 | 0.0002* | 0.336 ± 0.112 | <0.0001* | 0.371 ± 0.090 | <0.0001* |
| **Hipp-Sens** | 0.453 ± 0.106 | 0.0002* | 0.379 ± 0.078 | <0.0001* | 0.397 ± 0.060 | <0.0001* |
| **Hipp-LCN** | 0.325 ± 0.063 | <0.0001* | 0.261 ± 0.086 | <0.0001* | 0.235 ± 0.064 | <0.0001* |
| **Hipp-SubC** | 0.202 ± 0.042 | <0.0001* | 0.146 ± 0.048 | <0.0001* | 0.163 ± 0.043 | 0.0002* |
| **Sens-Sens** | 0.590 ± 0.136 | 0.0002* | 0.504 ± 0.052 | <0.0001* | 0.506 ± 0.114 | <0.0001* |
| **Sens-LCN** | 0.414 ± 0.086 | <0.0001* | 0.345 ± 0.062 | <0.0001* | 0.311 ± 0.120 | 0.0006* |
| **Sens-SubC** | 0.237 ± 0.039 | <0.0001* | 0.165 ± 0.028 | <0.0001* | 0.189 ± 0.063 | 0.0002* |
| **LCN-LCN** | 0.579 ± 0.125 | <0.0001* | 0.445 ± 0.124 | <0.0001* | 0.380 ± 0.128 | 0.0004* |
| **LCN-SubC** | 0.208 ± 0.036 | <0.0001* | 0.139 ± 0.040 | <0.0001* | 0.142 ± 0.062 | 0.0002* |
| **SubC-SubC** | 0.160 ± 0.029 | <0.0001* | 0.110 ± 0.029 | <0.0001* | 0.130 ± 0.042 | 0.0010* |

**Supplementary Table 4.** Statistical outcomes Network-based FC analysis for each within and between network zFC. In case of a non-significant sex*treatment interaction. a two-way ANOVA was performed with main effects (sex. treatment) only.

|  | **Two-way ANOVA** | | | | | | | | | |
| --- | --- | --- | --- | --- | --- | --- | --- | --- | --- | --- |
| **Network** |  | **DF** | **Sum of squares** | **F ratio** | **P-value** |  | **DF** | **Sum of squares** | **F ratio** | **P-value** |
| **DMLN-DMLN** | *Sex*treatment* | 2 | 0.080 | 1.658 | 0.2074 | *Sex* | 1 | 0.022 | 0.866 | 0.3588 |
|  |  |  |  |  |  | *Treatment* | 2 | 0.107 | 2.110 | 0.1377 |
| **DMLN-Hipp** | *Sex*treatment* | 2 | 0.023 | 1.436 | 0.2554 | *Sex* | 1 | 0.169 | 2.028 | 1.650 |
|  |  |  |  |  |  | *Treatment* | 2 | 0.119 | 7.147 | 0.0030* |
| **DMLN-Sens** | *Sex*treatment* | 2 | 0.043 | 1.225 | 0.3075 | *Sex* | 1 | 0.022 | 1.228 | 0.2757 |
|  |  |  |  |  |  | *Treatment* | 2 | 0.090 | 2.508 | 0.0968 |
| **DMLN-LCN** | *Sex*treatment* | 2 | 0.060 | 3.862 | 0.0326* |  |  |  |  |  |
| **DMLN-SubC** | *Sex*treatment* | 2 | 0.020 | 5.243 | 0.0116* |  |  |  |  |  |
| **Hipp-Hipp** | *Sex*treatment* | 2 | 0.008 | 0.671 | 0.5194 | *Sex* | 1 | 0.018 | 3.032 | 0.0922 |
|  |  |  |  |  |  | *Treatment* | 2 | 0.084 | 6.869 | 0.0036* |
| **Hipp-Sens** | *Sex*treatment* | 2 | 0.011 | 0.776 | 0.4698 | *Sex* | 1 | 0.014 | 1.972 | 0.1704 |
|  |  |  |  |  |  | *Treatment* | 2 | 0.094 | 6.214 | 0.0055* |
| **Hipp-LCN** | *Sex*treatment* | 2 | 0.021 | 2.075 | 0.1432 | *Sex* | 1 | 0.000 | 0.087 | 0.7694 |
|  |  |  |  |  |  | *Treatment* | 2 | 0.037 | 3.360 | 0.0474* |
| **Hipp-SubC** | *Sex*treatment* | 2 | 0.011 | 4.242 | 0.0250* |  |  |  |  |  |
| **Sens-Sens** | *Sex*treatment* | 2 | 0.036 | 1.042 | 0.3647 | *Sex* | 1 | 0.004 | 0.240 | 0.6271 |
|  |  |  |  |  |  | *Treatment* | 2 | 0.088 | 2.481 | 0.0991 |
| **Sens-LCN** | *Sex*treatment* | 2 | 0.033 | 1.593 | 0.2199 | *Sex* | 1 | 0.000 | 0.016 | 0.8972 |
|  |  |  |  |  |  | *Treatment* | 2 | 0.044 | 2.029 | 0.1480 |
| **Sens-SubC** | *Sex*treatment* | 2 | 0.008 | 1.870 | 0.1728 | *Sex* | 1 | 0.005 | 2.303 | 0.1395 |
|  |  |  |  |  |  | *Treatment* | 2 | 0.026 | 5.764 | 0.0076* |
| **LCN-LCN** | *Sex*treatment* | 2 | 0.094 | 3.044 | 0.0621 | *Sex* | 1 | < 0.0001 | 0.000 | 0.9955 |
|  |  |  |  |  |  | *Treatment* | 2 | 0.071 | 2.048 | 0.1451 |
| **LCN-SubC** | *Sex*treatment* | 2 | 0.012 | 3.079 | 0.0608 | *Sex* | 1 | 0.000 | 0.257 | 0.6152 |
|  |  |  |  |  |  | *Treatment* | 2 | 0.017 | 3.768 | 0.0339* |
| **SubC-SubC** | *Sex*treatment* | 2 | 0.007 | 4.393 | 0.0215* |  |  |  |  |  |

**Supplementary Table 5.** Statistical post-hoc (Tukey HSD) outcomes of network-based zFC analysis for each within and between network zFC. showing a significant treatment main effect.

|  | **Pairwise comparisons Tukey HSD (treatment)** | | | | | | |
| --- | --- | --- | --- | --- | --- | --- | --- |
| **Network** | **Treatment** | **Difference** | **Std Error** | **t-value** | **P-value** | **Lower 95%** | **Upper 95%** |
| **DMLN-Hipp** | *CR-Ctrl* | -0.076 | 0.039 | -1.91 | 0.1545 | -0.174 | 0.022 |
|  | *CR-Rsv* | 0.071 | 0.038 | 1.88 | 0.1637 | -0.022 | 0.165 |
|  | *Ctrl-Rsv* | 0.147 | 0.039 | 3.78 | 0.0020* | 0.051 | 0.244 |
| **DMLN-SubC** | *CR-Ctrl* | -0.035 | 0.024 | -1.47 | 0.3175 | -0.095 | 0.023 |
|  | *CR-Rsv* | 0.032 | 0.023 | 1.36 | 0.3722 | -0.026 | 0.090 |
|  | *Ctrl-Rsv* | 0.067 | 0.024 | 2.81 | 0.0226* | 0.008 | 0.127 |
| **Hipp-Hipp** | *CR-Ctrl* | -0.068 | 0.034 | -2.01 | 0.1289 | -0.153 | 0.015 |
|  | *CR-Rsv* | 0.055 | 0.032 | 1.70 | 0.2222 | -0.025 | 0.136 |
|  | *Ctrl-Rsv* | 0.124 | 0.033 | 3.71 | 0.0025* | 0.041 | 0.207 |
| **Hipp-Sens** | *CR-Ctrl* | -0.054 | 0.037 | -1.45 | 0.3269 | -0.146 | 0.037 |
|  | *CR-Rsv* | 0.075 | 0.035 | 2.12 | 0.1023 | -0.012 | 0.163 |
|  | *Ctrl-Rsv* | 0.129 | 0.037 | 3.49 | 0.0043* | 0.038 | 0.221 |
| **Hipp-LCN** | *CR-Ctrl* | -0.037 | 0.031 | -1.21 | 0.4569 | -0.114 | 0.038 |
|  | *CR-Rsv* | 0.041 | 0.030 | 1.38 | 0.3608 | -0.032 | 0.115 |
|  | *Ctrl-Rsv* | 0.079 | 0.030 | 2.59 | 0.0375* | 0.003 | 0.154 |
| **Hipp-SubC** | CR-Ctrl | -0.036 | 0.018 | -2.02 | 0.1271 | -0.081 | 0.008 |
|  | CR-Rsv | 0.030 | 0.017 | 1.81 | 0.1848 | -0.011 | 0.072 |
|  | Ctrl-Rsv | 0.067 | 0.017 | 3.86 | 0.0017* | 0.024 | 0.110 |
| **LCN-SubC** | CR-Ctrl | -0.025 | 0.020 | -1.24 | 0.4404 | -0.074 | 0.024 |
|  | CR-Rsv | 0.029 | 0.019 | 1.51 | 0.3014 | -0.018 | 0.077 |
|  | Ctrl-Rsv | 0.054 | 0.019 | 2.73 | 0.0266* | 0.005 | 0.103 |
| **Sens-SubC** | CR-Ctrl | -0.026 | 0.020 | -1.29 | 0.4131 | -0.076 | 0.024 |
|  | CR-Rsv | 0.041 | 0.019 | 2.14 | 0.0986 | -0.006 | 0.089 |
|  | Ctrl-Rsv | 0.068 | 0.020 | 3.34 | 0.0062* | 0.017 | 0.118 |

**Supplementary Table 6.** Statistical post-hoc (Student t-test) outcomes of network-based zFC analysis for each within and between network zFC. showing a significant sex*treatment interaction effect.

|  | **Pairwise comparisons Student T-test (sex*treatment)** | | | | | | | | |
| --- | --- | --- | --- | --- | --- | --- | --- | --- | --- |
| **Network** | **Treatment** | **Difference** | **Std Error** | **t value** | **P-value** | **FDR correction** | **Lower 95%** | **Upper 95%** |  |
| **DMLN-LCN** | Female CR - Female Ctrl | -0.114 | 0.049 | -2.33 | 0.0272* | 0.0816 | -0.215 | -0.013 |  |
|  | Female CR – Female Rsv | -0.041 | 0.047 | -0.88 | 0.3858 | 0.3858 | -0.138 | 0.055 |  |
|  | Female CR – Male CR | -0.056 | 0.051 | -1.08 | 0.2878 | 0.3263 | -0.162 | 0.049 |  |
|  | Female Ctrl – Female Rsv | 0.072 | 0.049 | 1.48 | 0.1497 | 0.2694 | -0.027 | 0.173 |  |
|  | Female Ctrl – Male Ctrl | 0.122 | 0.057 | 2.14 | 0.0407* | 0.0916 | 0.005 | 0.239 |  |
|  | Female Rsv – Male Rsv | 0.120 | 0.049 | 2.45 | 0.0206* | 0.0816 | 0.019 | 0.221 |  |
|  | Male CR – Male Ctrl | 0.064 | 0.059 | 1.08 | 0.2900 | 0.3263 | -0.057 | 0.185 |  |
|  | Male CR – Male Rsv | 0.135 | 0.053 | 2.52 | 0.0175* | 0.0816 | 0.025 | 0.244 |  |
|  | Male Ctrl – Male Rsv | 0.071 | 0.057 | 1.24 | 0.2236 | 0.3263 | -0.045 | 0.187 |  |
| **Hipp-SubC** | Female CR - Female Ctrl | -0.069 | 0.021 | -3.17 | 0.0038* | 0.019* | -0.114 | -0.024 |  |
|  | Female CR – Female Rsv | 0.017 | 0.020 | 0.83 | 0.4133 | 0.4769 | -0.025 | 0.060 |  |
|  | Female CR – Male CR | -0.020 | 0.021 | -0.94 | 0.3533 | 0.4769 | -0.065 | 0.024 |  |
|  | Female Ctrl – Female Rsv | 0.087 | 0.021 | 3.96 | 0.0005* | 0.0038* | 0.041 | 0.132 |  |
|  | Female Ctrl – Male Ctrl | 0.068 | 0.022 | 2.97 | 0.0061* | 0.0229* | 0.021 | 0.115 |  |
|  | Female Rsv – Male Rsv | 0.000 | 0.020 | 0.04 | 0.9667 | 0.9667 | -0.042 | 0.043 |  |
|  | Male CR – Male Ctrl | 0.019 | 0.022 | 0.85 | 0.4051 | 0.4769 | -0.027 | 0.066 |  |
|  | Male CR – Male Rsv | 0.039 | 0.021 | 1.78 | 0.0869 | 0.201 | -0.006 | 0.084 |  |
|  | Male Ctrl – Male Rsv | 0.019 | 0.021 | 0.89 | 0.3795 | 0.4769 | -0.025 | 0.064 |  |
| **LCN-SubC** | Female CR - Female Ctrl | -0.089 | 0.029 | -3.09 | 0.0043* | 0.0193* | -0.148 | -0.030 |  |
|  | Female CR – Female Rsv | 0.012 | 0.029 | 0.42 | 0.6769 | 0.7615 | -0.047 | 0.071 |  |
|  | Female CR – Male CR | -0.025 | 0.030 | -0.84 | 0.4070 | 0.6663 | -0.088 | 0.036 |  |
|  | Female Ctrl – Female Rsv | 0.101 | 0.030 | 3.38 | 0.0020* | 0.0180* | 0.040 | 0.163 |  |
|  | Female Ctrl – Male Ctrl | 0.089 | 0.031 | 2.84 | 0.0081* | 0.0243* | 0.025 | 0.154 |  |
|  | Female Rsv – Male Rsv | 0.001 | 0.029 | 0.03 | 0.9798 | 0.9798 | -0.058 | 0.060 |  |
|  | Male CR – Male Ctrl | 0.025 | 0.033 | 0.78 | 0.4442 | 0.6663 | -0.041 | 0.092 |  |
|  | Male CR – Male Rsv | 0.038 | 0.030 | 1.27 | 0.2156 | 0.4851 | -0.023 | 0.101 |  |
|  | Male Ctrl – Male Rsv | 0.013 | 0.030 | 0.43 | 0.6720 | 0.7615 | -0.049 | 0.075 |  |
| **SubC** | Female CR - Female Ctrl | -0.052 | 0.017 | -2.96 | 0.006* | 0.0225* | -0.089 | -0.016 |  |
|  | Female CR – Female Rsv | 0.001 | 0.016 | 0.1 | 0.9201 | 0.9201 | -0.031 | 0.035 |  |
|  | Female CR – Male CR | -0.018 | 0.017 | -1.06 | 0.2984 | 0.373 | -0.055 | 0.017 |  |
|  | Female Ctrl – Female Rsv | 0.054 | 0.017 | 3.16 | 0.0037* | 0.0225* | 0.019 | 0.089 |  |
|  | Female Ctrl – Male Ctrl | 0.057 | 0.018 | 3.07 | 0.0046* | 0.0225* | 0.019 | 0.095 |  |
|  | Female Rsv – Male Rsv | 0.022 | 0.015 | 1.45 | 0.1587 | 0.2976 | -0.009 | 0.054 |  |
|  | Male CR – Male Ctrl | 0.023 | 0.018 | 1.25 | 0.2213 | 0.3476 | -0.014 | 0.061 |  |
|  | Male CR – Male Rsv | 0.043 | 0.017 | 2.51 | 0.0178* | 0.0534 | 0.008 | 0.078 |  |
|  | Male Ctrl – Male Rsv | 0.020 | 0.017 | 1.16 | 0.2549 | 0.3476 | -0.015 | 0.055 |  |
| **DMLN-SubC** | Female CR - Female Ctrl | -0.097 | 0.026 | -3.65 | 0.0011* | 0.0083* | -0.151 | -0.042 |  |
|  | Female CR – Female Rsv | -0.023 | 0.025 | -0.91 | 0.3691 | 0.5033 | -0.075 | 0.028 |  |
|  | Female CR – Male CR | -0.038 | 0.026 | -1.45 | 0.1593 | 0.2655 | -0.092 | 0.016 |  |
|  | Female Ctrl – Female Rsv | 0.073 | 0.026 | 2.78 | 0.0097* | 0.0364* | 0.019 | 0.128 |  |
|  | Female Ctrl – Male Ctrl | 0.079 | 0.027 | 2.86 | 0.0079* | 0.0364* | 0.022 | 0.136 |  |
|  | Female Rsv – Male Rsv | 0.050 | 0.024 | 2.06 | 0.0491* | 0.1052 | 0.002 | 0.100 |  |
|  | Male CR – Male Ctrl | 0.020 | 0.027 | 0.75 | 0.4573 | 0.5716 | -0.035 | 0.077 |  |
|  | Male CR – Male Rsv | 0.065 | 0.025 | 2.55 | 0.0165* | 0.0495* | 0.012 | 0.118 |  |
|  | Male Ctrl – Male Rsv | 0.044 | 0.025 | 1.74 | 0.0936 | 0.1755 | -0.008 | 0.097 |  |


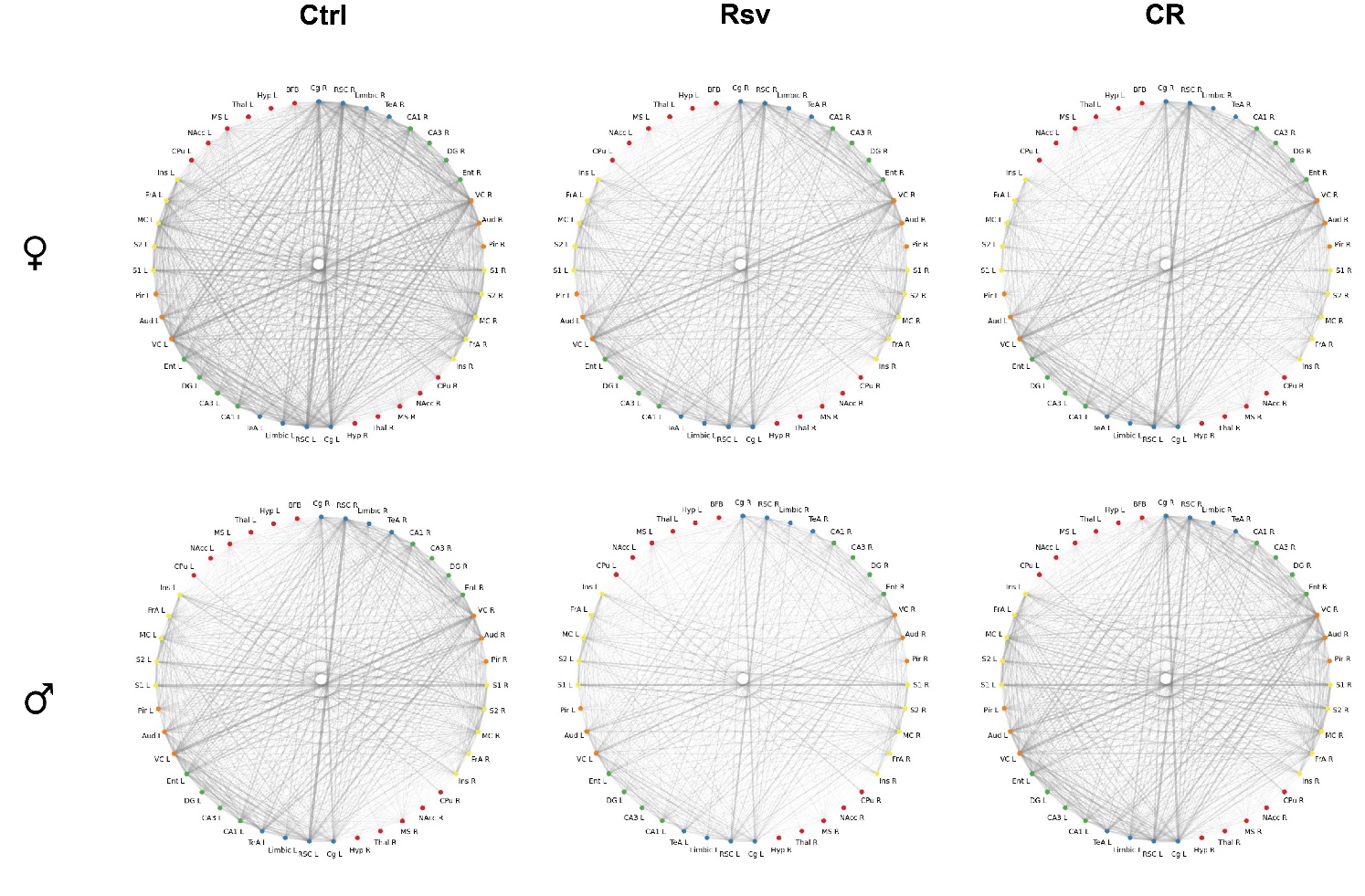


**Supplementary Figure 1.** FC in male and female Ctrl, Rsv supplemented and CR rats. Chord plots displaying the mean FC between ROI pairs for males and females per treatment group (Ctrl = control, CR = caloric restriction, Rsv = Resveratrol. The width and transparency of the edges indicate the strength of FC. Broad and dark grey edges indicate high FC and narrow light grey edges indicate low FC. Non-significant connections (p > 0.05, one-sample t-test, FDR corrected per group) are not shown. The colours of the nodes indicate the corresponding RSNs of the ROIs: DMLN (default mode-like network = blue), Hipp (hippocampal network = green), Sens (sensory network= orange), LCN (lateral cortical network = yellow), SubC (subcortical network = red).
